# Perinatal Changes in Serum Fibroblast Growth Factor 21 and Their Association With Postpartum Insulin Resistance in Women With Gestational Diabetes Mellitus

**DOI:** 10.1111/jog.70480

**Published:** 2026-08-30

**Authors:** Yosuke Suzuki, Akihito Morita, Jun Okaniwa, Ayuko Tanaka, Yuko Hasegawa, Tatsuya Sato, Daisuke Higeta, Akira Iwase

**Affiliations:** ^1^ Department of Obstetrics and Gynecology Gunma University Graduate School of Medicine Maebashi Gunma Japan; ^2^ Department of Obstetrics and Gynecology Tone Chuo Hospital Numata Gunma Japan; ^3^ Department of Medical Education and Development Gunma University Graduate School of Medicine Maebashi Gunma Japan

**Keywords:** fibroblast growth factor 21, gestational diabetes, insulin resistance, longitudinal survey, postpartum

## Abstract

**Aim:**

This study aimed to clarify the clinical significance of perinatal changes in fibroblast growth factor 21 and their association with postpartum metabolic outcomes in women with gestational diabetes mellitus.

**Methods:**

This single‐center retrospective observational study used prospectively collected residual serum samples to investigate longitudinal changes in serum fibroblast growth factor 21 concentrations at approximately 26 (T1) and 36 (T2) weeks of gestation and within 7 days postpartum (T3) in 45 women with gestational diabetes mellitus and 30 controls. Associations between fibroblast growth factor 21 concentrations and postpartum metabolic outcomes were evaluated in the gestational diabetes mellitus group.

**Results:**

Serum fibroblast growth factor 21 concentrations increased significantly from T1 to T2 and remained elevated at T3, irrespective of gestational diabetes mellitus status, as assessed using the Friedman test with post hoc comparisons. In women with gestational diabetes mellitus, fibroblast growth factor 21 showed predictive performance for postpartum insulin resistance, as assessed using the homeostasis model assessment of insulin resistance. Receiver operating characteristic analysis showed the highest area under the curve for predicting postpartum insulin resistance at T1 (area under the curve, 0.871; bootstrap 95% confidence interval, 0.732–0.973). Fibroblast growth factor 21 concentrations appeared to decline during the early postpartum period, as indicated by significantly lower T3/T1 ratios in samples from postpartum Days 5–7 compared with those from Days 1–4.

**Conclusions:**

These findings suggest that perinatal fibroblast growth factor 21 dynamics may reflect physiological changes in insulin sensitivity and that fibroblast growth factor 21 concentrations measured during pregnancy may serve as a complementary indicator for postpartum metabolic risk stratification in women with gestational diabetes mellitus. Further validation is needed before clinical application.

## 1 Introduction

Fibroblast growth factor 21 (FGF21) is a hepatokine primarily expressed in the liver that is crucial in regulating lipid and glucose metabolism [1]. Elevated circulating FGF21 levels have been reported in metabolic conditions characterized by insulin resistance, such as obesity and Type 2 diabetes mellitus, where it promotes glucose uptake and enhances insulin sensitivity [2, 3]. Given its metabolic regulatory functions, FGF21 has been suggested to be associated with gestational diabetes mellitus (GDM), an insulin‐resistant state.

GDM is typically diagnosed between 24 and 28 weeks of gestation using a 75 g oral glucose tolerance test (OGTT) [4]. FGF21 has been proposed as a biomarker for GDM, and studies have reported elevated circulating FGF21 levels in pregnant women with GDM [5, 6, 7, 8]. In contrast, other studies have found higher FGF21 levels in the control group [9] or reported no significant differences [10, 11, 12, 13]. Recent meta‐analyses have indicated that FGF21 levels are significantly higher in women with GDM than in controls and that heterogeneity is influenced by factors such as the sample size, geographical location, and timing of blood sampling [14, 15]. Importantly, most previous studies have assessed FGF21 at a single time point with variable timing (e.g., early, mid, or late pregnancy, or at delivery), resulting in poor comparability. Few studies have evaluated the longitudinal changes in FGF21 levels from pregnancy to the postpartum period within the same individuals [5, 9], although such data are crucial for understanding the dynamics of FGF21 in relation to maternal glucose metabolism.

Because GDM is associated with an increased risk of persistent glucose intolerance and future Type 2 diabetes mellitus, effective postpartum management is essential but often insufficient [16]. Internationally, the rate of postpartum OGTT among women with a history of GDM varies by region and institution and is often suboptimal [17, 18, 19, 20], partly due to the burden of the OGTT. Although several studies have suggested a relationship between FGF21 and insulin resistance during pregnancy [5, 6, 7], the associations between FGF21 concentrations measured during pregnancy and the early postpartum period and postpartum glucose intolerance or insulin resistance remain poorly understood.

This study aimed to clarify these associations and investigate the longitudinal changes in serum FGF21 concentrations during and after pregnancy in women with and without GDM. If confirmed, these associations would support the use of FGF21 as a biomarker for postpartum risk stratification in women with GDM.

## 2 Methods

### 2.1 Study Population

This was a single‐center, retrospective, observational study using prospectively collected residual serum samples. Serum samples were collected from pregnant women aged 20 years or older who delivered at Gunma University Hospital between February 2019 and July 2024 and who provided written informed consent. In accordance with the institutional clinical protocol, blood samples were collected at three clinically indicated time points during pregnancy and the early postpartum period, and residual serum samples were subsequently obtained. At T1 and T2, blood samples were obtained 2–4 h after meals as part of routine non‐fasting clinical testing, whereas T3 samples were obtained under fasting conditions. Using medical records, we identified women for whom preserved serum samples were available at all three time points and for whom the remaining serum volume was sufficient for FGF21 measurement. From this eligible population, we selected 45 women with GDM and 30 women without GDM as controls without applying a formal randomization procedure. More participants were included in the GDM group than in the control group to enable subsequent analyses of the association between FGF21 concentrations and postpartum metabolic outcomes within the GDM group. The number of controls was determined by the availability of residual serum samples with sufficient volume and the feasibility of FGF21 measurement.

GDM screening was performed according to the routine clinical practice in Japan. In early pregnancy, random plasma glucose levels were measured, and women with elevated values underwent a 75 g OGTT. At approximately 24–28 weeks of gestation, women were screened using either random plasma glucose or a 50 g glucose challenge test, and those with abnormal results underwent a 75 g OGTT. GDM was diagnosed according to the criteria of the International Association of Diabetes and Pregnancy Study Groups: fasting plasma glucose level ≥ 92 mg/dL (5.1 mmol/L), a 1‐h value ≥ 180 mg/dL (10.0 mmol/L), or a 2‐h value ≥ 153 mg/dL (8.5 mmol/L) during the 75 g OGTT [4]. All patients with GDM were referred to certified diabetologists for dietary therapy and self‐monitoring of blood glucose levels. Insulin therapy was initiated if the blood glucose control was insufficient, such as when the target glycemic levels (e.g., a 2‐h postprandial glucose level of < 120 mg/dL [6.7 mmol/L]) could not be achieved with dietary therapy alone.

Women with preexisting diabetes mellitus, systemic corticosteroid use during pregnancy, preterm delivery, or fetal anomalies identified during pregnancy were excluded from the study.

For all 75 participants, the following maternal characteristics and perinatal outcomes were extracted from medical records: maternal age, obstetric history, history of GDM, family history of diabetes mellitus, use of assisted reproductive technology, pre‐pregnancy body mass index (BMI), gestational weight gain, gestational age at delivery, birth weight, mode of delivery, shoulder dystocia, neonatal sex, neonatal hypoglycemia, neonatal jaundice, Apgar scores, umbilical artery blood pH, and admission to the neonatal intensive care unit.

In the GDM group, we additionally recorded the gestational week at diagnosis, 75 g OGTT and hemoglobin A1c (HbA1c) levels at the time of diagnosis, use of insulin during pregnancy, and dosage at delivery. Among the 45 women with GDM, 36 underwent a postpartum 75 g OGTT and fasting insulin measurements between 6 and 12 weeks postpartum. These data were used to evaluate postpartum glucose intolerance and insulin resistance and to perform receiver operating characteristic (ROC) and subgroup analyses of postpartum metabolic outcomes.

### 2.2 Sample Collection and FGF21 Assay

Blood samples were collected at three time points. The sampling times were as follows: for T1, the median gestational age was 26 weeks (interquartile range [IQR]: 25–27 weeks; range: 24–32 weeks) and for T2, the median was 36 weeks (IQR: 35–36 weeks; range: 34–37 weeks). At T3, the median postpartum period was 3 days (IQR: 2–3 days; range: 1–5 days) for women who underwent vaginal delivery and 7 days (IQR: 6–7 days; range: 3–7 days) for those who underwent a cesarean section.

Serum was separated and stored at −80°C until analysis. Serum FGF21 concentrations were measured using a commercially available enzyme immunoassay kit (human FGF21 ELISA Kit [ab222506]; Abcam Ltd., Cambridge, UK).

### 2.3 Statistical Analysis

Background characteristics were compared between the GDM and control groups using the Mann–Whitney U test for continuous variables and the Fisher's exact test for categorical variables. Absolute serum FGF21 concentrations were additionally compared between the GDM and control groups at each time point using the Mann–Whitney U test.

Within‐group changes in serum FGF21 concentrations across the three time points (T1–T3) were assessed using the Friedman test for each group (GDM and control). When the Friedman test indicated statistical significance, post hoc pairwise comparisons between time points (T1–T2, T1–T3, and T2–T3) were conducted using the Wilcoxon signed‐rank test. To adjust for multiple comparisons, *p*‐values from the Wilcoxon tests were corrected using the Benjamini and Hochberg method to calculate the false discovery rate (FDR). As a sensitivity analysis to assess the potential influence of pre‐pregnancy BMI, the longitudinal changes in serum FGF21 concentrations were additionally analyzed after restricting the cohort to participants with a pre‐pregnancy BMI < 25 kg/m^2^.

To examine the effect of postpartum sampling timing on FGF21 dynamics, the ratios of FGF21 concentrations at T3 relative to those at T1 and T2 were calculated and transformed using the log1p function [i.e., log(1 + x)]. Participants were categorized into early (postpartum Days 1–4) and late (Days 5–7) subgroups, and the transformed ratios were compared between the groups using the Mann–Whitney U test. Spearman's rank correlation coefficients were calculated to assess the association between the postpartum day (as a continuous variable) and the log1p‐transformed T3/T1 and T3/T2 ratios in the overall cohort and within each group. The relationship between the postpartum day and transformed ratios was visualized using scatter plots and linear regression lines.

For the 36 participants with GDM who underwent postpartum OGTT, ROC analysis was conducted to evaluate the predictive value of FGF21 concentrations at each time point for postpartum glucose intolerance and insulin resistance. Postpartum glucose intolerance was defined as a fasting glucose level ≥ 110 mg/dL (6.1 mmol/L) or a 2‐h glucose level ≥ 140 mg/dL (7.8 mmol/L) according to the diagnostic criteria of the Japan Diabetes Society. Insulin resistance was defined as a homeostasis model assessment of insulin resistance (HOMA‐IR) ≥ 2.5 [21]. The area under the curve (AUC) was calculated, and optimal cutoff values were determined using the Youden index. In addition, the sensitivity, specificity, positive predictive value (PPV), and negative predictive value (NPV) were computed for each cutoff to evaluate the diagnostic performance for both glucose intolerance and insulin resistance. The 95% confidence intervals (CIs) for the AUCs were estimated using stratified nonparametric bootstrap resampling with 1000 iterations.

Additional subgroup analyses were performed within the GDM group according to insulin use during pregnancy and the timing of GDM diagnosis, categorized as before 24 weeks 0 days of gestation or at 24 weeks 0 days of gestation or later. Serum FGF21 concentrations and postpartum metabolic outcomes were compared between subgroups using the Mann–Whitney U test for continuous variables and Fisher's exact test for categorical variables.

Statistical significance was set at *p* < 0.05. All statistical analyses were performed using Microsoft Excel 2019 and EZR (Saitama Medical Center, Jichi Medical University), which is a graphical user interface for R (R Foundation for Statistical Computing, Vienna, Austria) [22]. All Figures were generated using Python 3.11 with the Matplotlib and seaborn libraries.

## 3 Results

### 3.1 Patient Characteristics

The patient characteristics are summarized in Table 1. All participants were Japanese. The maternal age, gestational age at delivery, and rates of cesarean section or assisted vaginal delivery were not significantly different between the GDM and control groups. The neonatal birth weight and perinatal outcomes were comparable between the two groups.

**TABLE 1:** Comparisons of maternal and neonatal characteristics between GDM group and control group.

	Control (*n* = 30)	GDM (*n* = 45)	*p*
*Maternal characteristics*			
Maternal age (years)	35 (30.5–37)	33 (29–38)	0.389
Family history of DM, *n* (%)	8 (27)	17 (38)	0.454
History of GDM, *n* (%)	0 (0)	5 (11)	0.079
ART pregnancy, *n* (%)	4 (13)	8 (18)	0.752
Multiparity, *n* (%)	15 (50)	19 (42)	0.637
Pre‐pregnancy BMI (kg/m^2^)	21.5 (19.6–23.0)	25.9 (22.1–31.2)	< 0.001
Pre‐pregnancy BMI ≥ 25 kg/m^2^, *n* (%)	4 (13)	25 (56)	< 0.001
Pre‐pregnancy BMI ≥ 30 kg/m^2^, *n* (%)	0 (0)	13 (29)	0.001
Gestational weight gain (kg)	10.8 (7.6–12.6)	7.5 (4.5–11)	0.004
*Delivery outcomes*			
Gestational age at delivery (weeks)	38.9 (38.5–39.4)	38.9 (38.4–39.3)	0.622
Cesarean section delivery, *n* (%)	12 (40)	18 (40)	1
Emergency cesarean delivery, *n* (%)	5 (17)	5 (11)	0.508
Forced delivery, *n* (%)	2 (7)	2 (4)	1
Shoulder dystocia, *n* (%)	1 (3)	0 (0)	0.4
*Neonatal outcomes*			
Birth weight (g)	2996 (2845–3170)	3132 (2926–3308)	0.165
LGA, *n* (%)	4 (13)	7 (16)	1
SGA, *n* (%)	1 (3)	1 (2)	1
Neonatal sex (female)	13 (43)	11 (24)	0.129
Neonatal hypoglycemia, *n* (%)	1 (3)	2 (4)	1
Neonatal jaundice, *n* (%)	5 (17)	9 (20)	0.772
Apgar score at 5 min ≤ 7, *n* (%)	2 (7)	0 (0)	0.157
UA pH < 7.25, *n* (%)	1 (3)	2 (4)	1
NICU admission, *n* (%)	3 (10)	4 (9)	1

*Note:* Data are presented as the median (interquartile range) or *n* (%). *p*‐values were calculated using the Mann–Whitney U test for continuous variables and Fisher's exact test for categorical variables.

Abbreviations: ART, assisted reproductive technology; BMI, body mass index; DM, diabetes mellitus; GDM, gestational diabetes mellitus; LGA, large‐for‐gestational age; NICU, neonatal intensive care unit; SGA, small‐for‐gestational age; UA, umbilical artery.

The pre‐pregnancy BMI was significantly higher in the GDM group (median [IQR]: 25.9 [22.1–31.2] kg/m^2^) than in the control group (21.5 [19.6–23.0] kg/m^2^). In the control group, four women (13%) had a BMI ≥ 25 kg/m^2^, and none had a BMI ≥ 30 kg/m^2^. In contrast, 25 women (56%) in the GDM group had a BMI ≥ 25 kg/m^2^, and 13 (29%) had a BMI ≥ 30 kg/m^2^, indicating a marked difference in the prevalence of overweight and obesity between the groups. A history of GDM was observed in five patients (11%) in the GDM group and in none of the patients in the control group; however, this difference was not significant (*p* = 0.079). The gestational weight gain was significantly lower in the GDM group (median [IQR]: 7.5 [4.5–11] kg) than in the control group (10.8 [7.6–12.6] kg; *p* < 0.01).

Among women in the GDM group, 13 (29%) were diagnosed at 24 weeks 0 days of gestation or later. Insulin therapy was required in 26 cases (58%), with a median total daily dose of 8 units (IQR: 2.5–42.5 units) at delivery. Based on the OGTT at diagnosis, glucose levels exceeded the diagnostic thresholds in 27 patients (60%) at fasting, 26 patients (58%) at 1 h, and 23 patients (51%) at 2 h. The median HbA1c level at diagnosis was 5.5% (IQR: 5.2%–5.6%), with no cases exceeding 6.5%. No participant had a fasting glucose level > 126 mg/dL (7.0 mmol/L).

### 3.2 Changes in Serum FGF21 Concentrations

**FIGURE 1:**
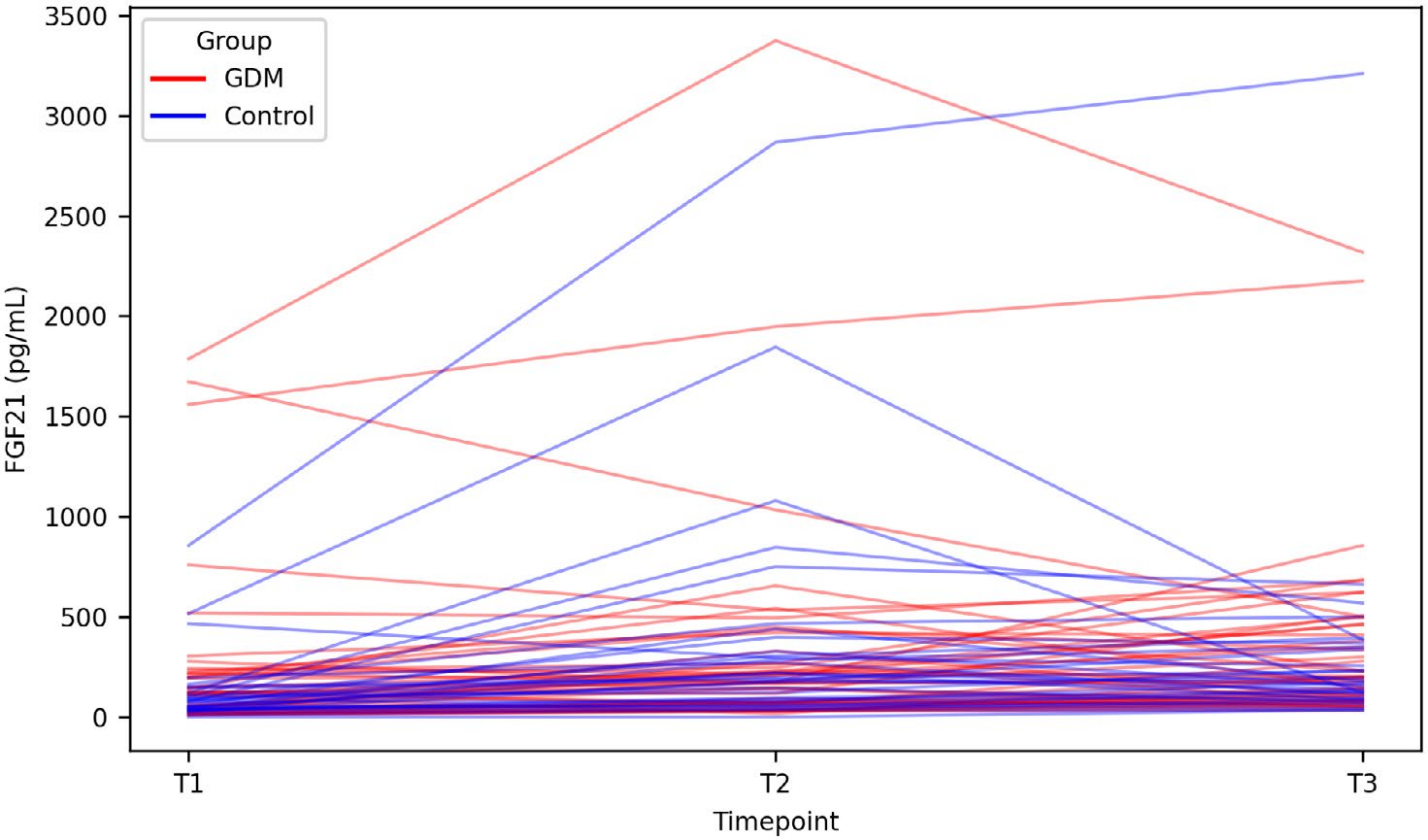
Individual trajectories of serum FGF21 concentrations during pregnancy and the early postpartum period. Individual trajectories of serum FGF21 concentrations at approximately 26 (T1) and 36 (T2) weeks of gestation and within 7 days postpartum (T3) in women with GDM (red) and in controls (blue). Each line represents one participant. FGF21, fibroblast growth factor 21; GDM, gestational diabetes mellitus.

**FIGURE 2:**
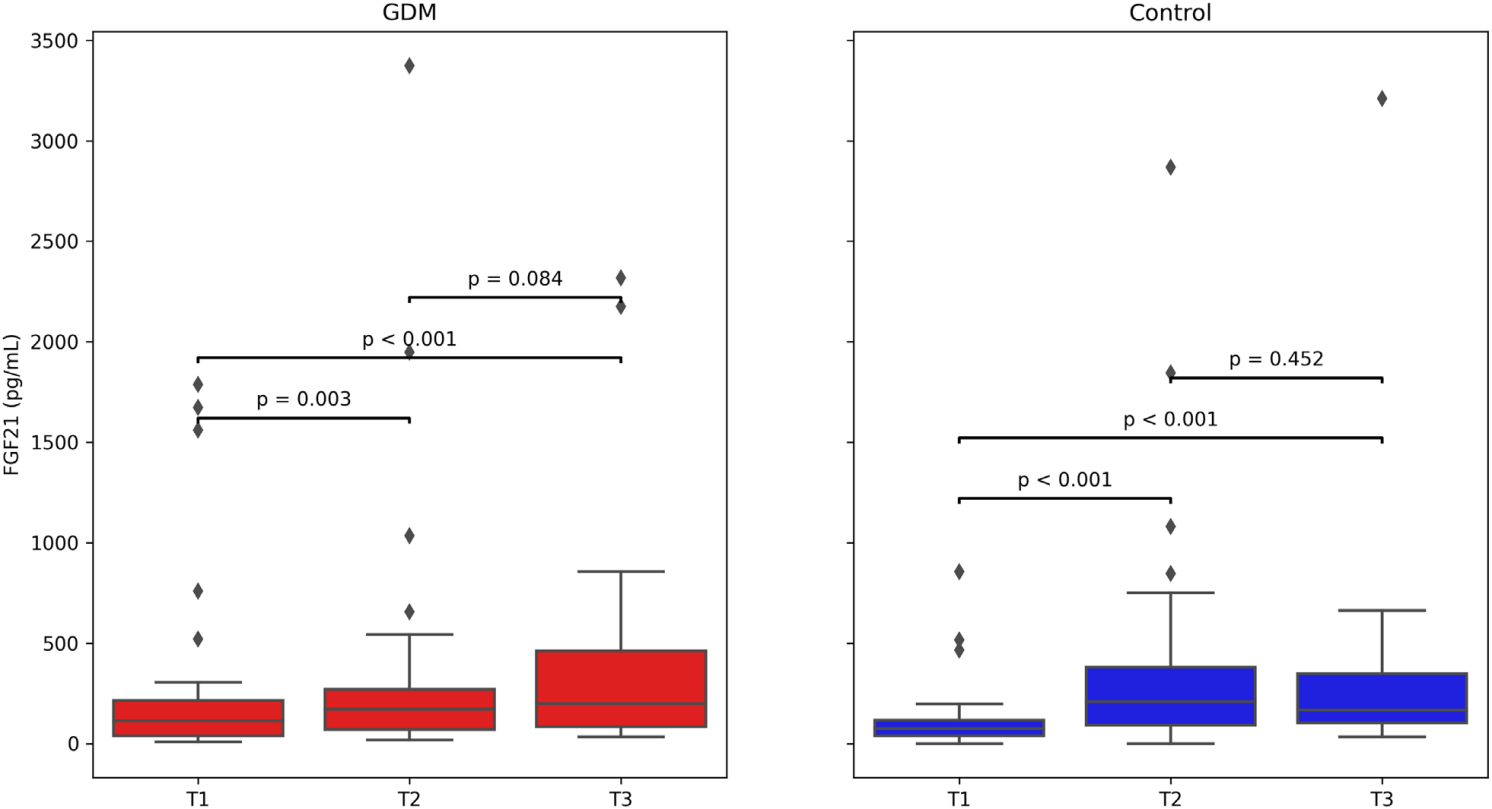
Longitudinal changes in serum FGF21 concentrations during pregnancy and the early postpartum period. Serum FGF21 concentrations were measured at approximately 26 (T1) and 36 (T2) weeks of gestation and within 7 days postpartum (T3) in women with GDM and controls. Box plots show median, IQR, and whiskers extending to 1.5 × IQR from the quartiles. *p*‐values are based on Wilcoxon signed‐rank tests with post hoc false discovery rate corrections based on significant Friedman test results. FGF21, fibroblast growth factor 21; GDM, gestational diabetes mellitus; IQR, interquartile range.

The line graph (Figure 1) shows an overall increase in serum FGF21 concentrations from T1 to T3 in many patients in the GDM and control groups. The boxplot (Figure 2) shows that the GDM group (*n* = 45) had a median (IQR) FGF21 concentration of 113 (39–214), 172 (70–269), and 198 (83–461) pg/mL at T1, T2, and T3, respectively. In the control group (*n* = 30), the median (IQR) concentrations were 75 (38–117), 208 (90–380), and 167 (102–347) at T1, T2, and T3, respectively. In direct between‐group comparisons, FGF21 concentrations did not differ significantly between the GDM and control groups at T1, T2, and T3 (*p* = 0.357, *p* = 0.258, and *p* = 0.580, respectively).

The Friedman test showed significant differences in serum FGF21 concentrations across the three time points in both groups (*p* < 0.001 for both). Post hoc analysis with Wilcoxon signed‐rank tests and FDR correction revealed significant differences between T1 and T2 (adjusted *p* = 0.003) and between T1 and T3 (adjusted *p* < 0.001) in the GDM group, whereas the difference between T2 and T3 was not significant (adjusted *p* = 0.084). In the control group, significant differences were found between T1 and T2 (adjusted *p* < 0.001) and between T1 and T3 (adjusted *p* < 0.001), but not between T2 and T3 (adjusted *p* = 0.452).

As a sensitivity analysis, the longitudinal changes in serum FGF21 concentrations were reanalyzed after restricting the cohort to participants with a pre‐pregnancy BMI < 25 kg/m^2^ (Figure S1). In the BMI‐restricted subgroup, serum FGF21 concentrations differed significantly over time in the GDM and control groups. In both groups, FGF21 concentrations were significantly higher at T2 and T3 than at T1, whereas no significant difference was observed between T2 and T3. This pattern was consistent with that observed in the full cohort.

**FIGURE 3:**
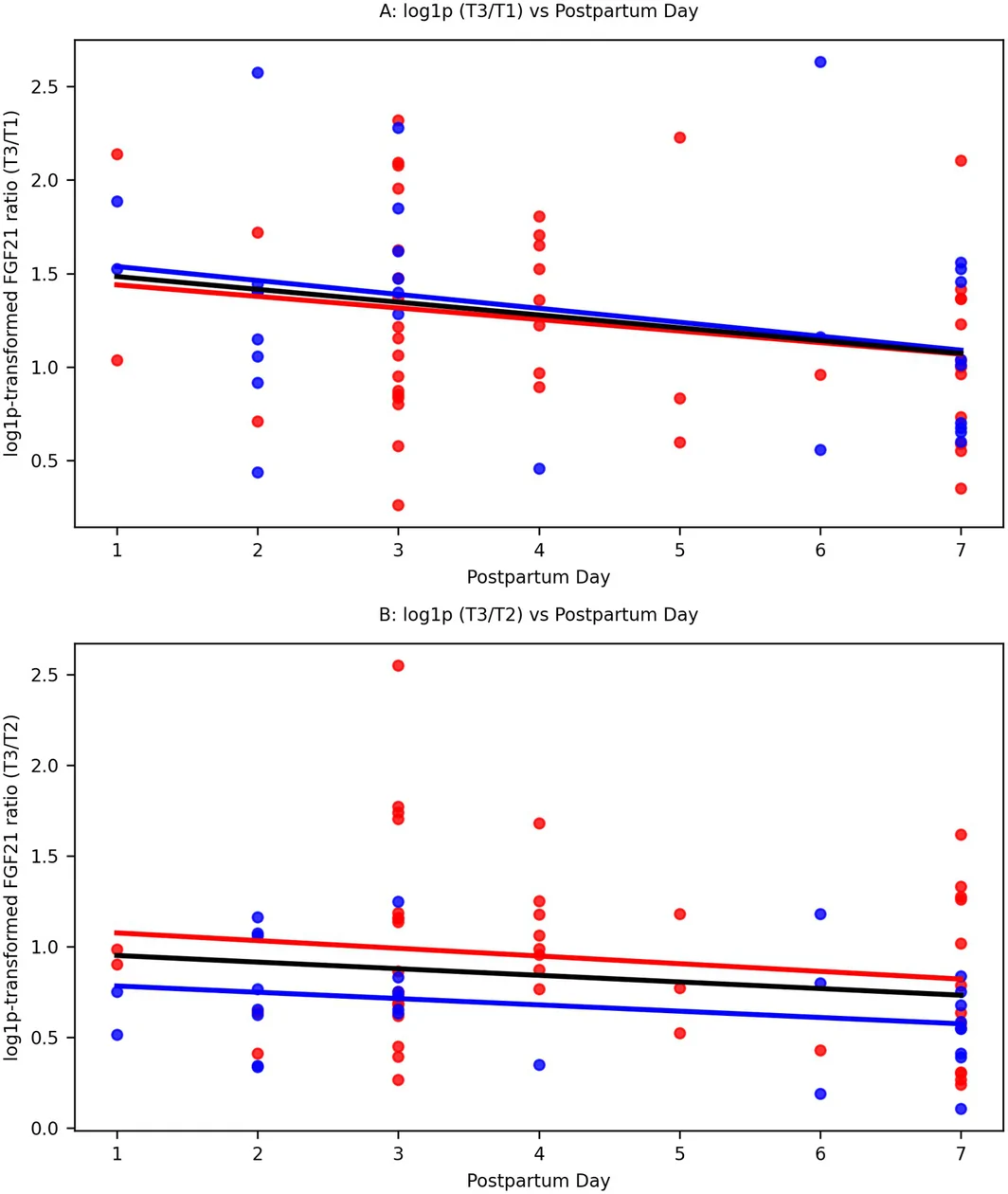
Association between postpartum sampling day and relative changes in serum FGF21 concentrations. Scatter plots showing the association between the postpartum day and log1p‐transformed FGF21 ratios. Each dot represents an individual (GDM: Red, control: Blue). Linear regression lines: Black (all), red (GDM), and blue (control). (A) T3/T1—All: *ρ* = −0.262, *p* = 0.023; GDM: *ρ* = −0.163, *p* = 0.286; control: *ρ* = −0.277, *p* = 0.138. (B) T3/T2—All: *ρ* = −0.129, *p* = 0.269; GDM: *ρ* = −0.078, *p* = 0.612; control: *ρ* = −0.111, *p* = 0.558. FGF21, fibroblast growth factor 21; GDM, gestational diabetes mellitus.

**FIGURE 4:**
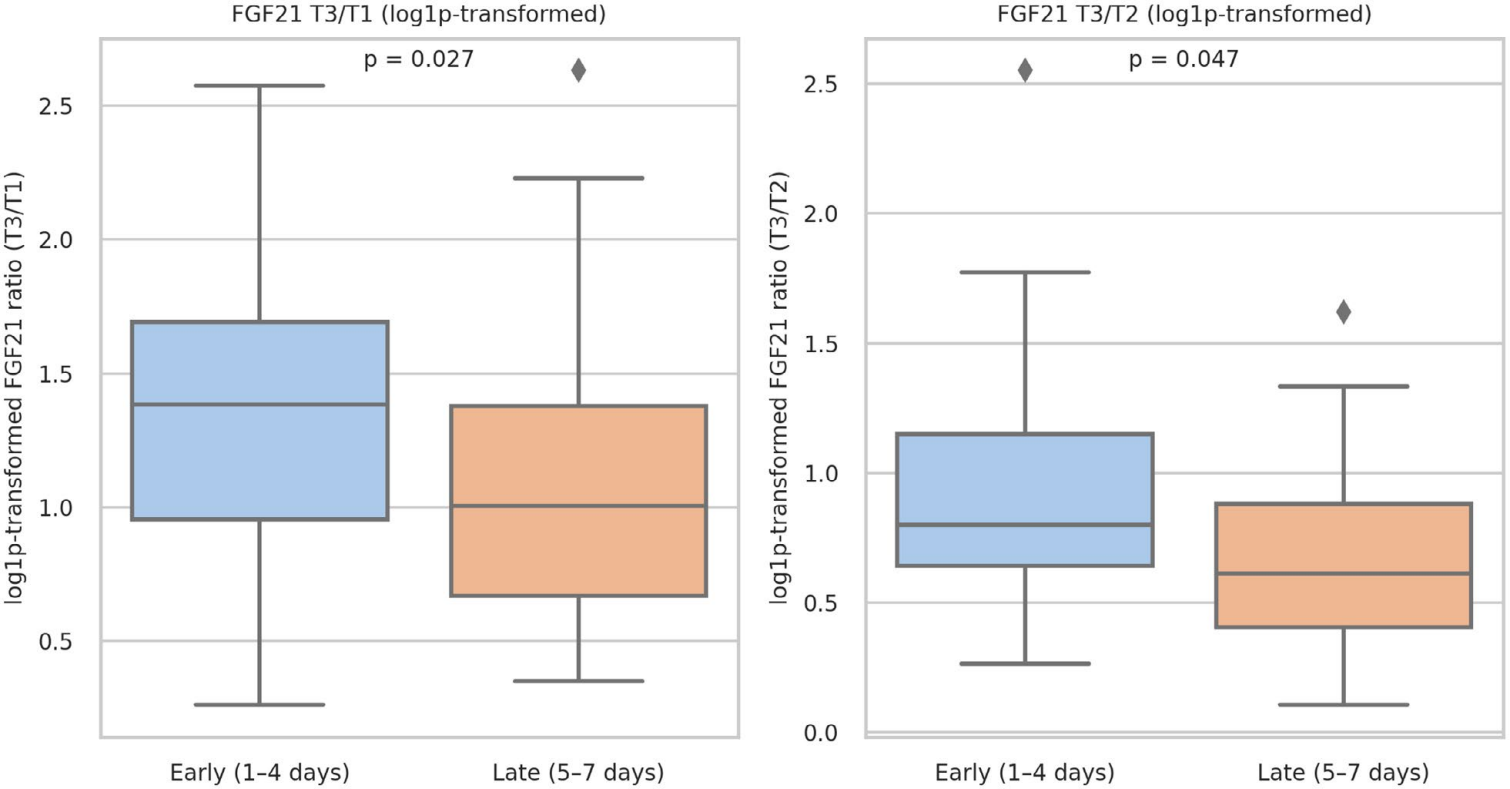
Relative postpartum FGF21 concentrations according to the timing of postpartum sampling. log1p‐transformed ratios of serum FGF21 concentrations within 7 days postpartum (T3) relative to those at approximately 26 (T1) and 36 (T2) weeks of gestation and comparisons between early (postpartum Days 1–4) and late (Days 5–7) sampling groups. Box plots show the median, IQR, and whiskers extending to 1.5 × IQR from the quartiles. *p*‐values were based on the Mann–Whitney U test. FGF21, fibroblast growth factor 21; IQR, interquartile range.

A scatterplot of the log1p‐transformed ratios of T3/T1 and T3/T2 against the number of postpartum days (Figure 3) showed a tendency toward lower ratios with later sampling dates. Spearman's rank correlation analysis revealed a significant negative correlation between postpartum day and the log1p‐transformed T3/T1 ratio in the overall cohort (*ρ* = −0.262, *p* = 0.023), whereas no significant associations were found for the T3/T2 ratio or in the subgroup analyses of the GDM and control groups. When participants were grouped according to the timing of blood collection (early, postpartum Days 1–4; late, Days 5–7), the log1p‐transformed T3/T1 and T3/T2 ratios were significantly lower in the late group (Figure 4). These findings were consistent with the decline in relative FGF21 concentrations with later postpartum sampling.

### 3.3 ROC Curves for Predicting Postpartum Glucose Intolerance and Insulin Resistance

**FIGURE 5:**
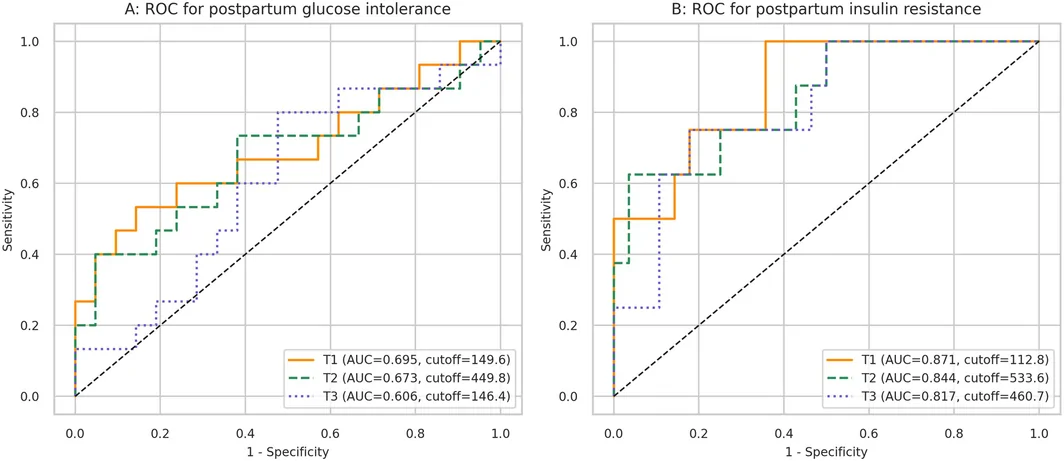
ROC curves of serum FGF21 concentrations for postpartum glucose intolerance and insulin resistance. ROC curves for FGF21 concentrations at approximately 26 (T1) and 36 (T2) weeks of gestation and within 7 days postpartum (T3) for the prediction of postpartum glucose intolerance (A) and insulin resistance (B) (HOMA‐IR ≥ 2.5) in women with GDM. Cutoff values were derived using the Youden index. See Results for the sensitivity, specificity, PPV, and NPV. AUC, area under the curve; FGF21, fibroblast growth factor 21; GDM, gestational diabetes mellitus; HOMA‐IR, homeostasis model assessment of insulin resistance; NPV, negative predictive value; PPV, positive predictive value.

Among the 45 women with GDM, 36 underwent postpartum OGTT and were included in the ROC analysis. Postpartum glucose intolerance was observed in 15 women, and insulin resistance, defined as HOMA‐IR ≥ 2.5, was observed in eight women. The ROC curves are shown in Figure 5. A summary of the AUCs with bootstrap 95% CIs, cutoff values, and diagnostic performance measures, including sensitivity, specificity, PPV, and NPV, are provided in Table 2.

**TABLE 2:** Diagnostic performance of FGF21 concentrations for predicting postpartum metabolic outcomes.

Timepoint	Outcome	AUC (bootstrap 95% CI)	Cutoff (pg/mL)	Sensitivity (%)	Specificity (%)	PPV (%)	NPV (%)
T1	Glucose intolerance	0.695 (0.498–0.867)	154.1	46.7	85.7	70	69.2
T2		0.673 (0.476–0.860)	202	53.3	66.7	53.3	66.7
T3		0.606 (0.419–0.790)	142.9	80	52.4	54.5	78.6
T1	Insulin resistance (HOMA‐IR ≥ 2.5)	0.871 (0.732–0.973)	175.7	62.5	82.1	50	88.5
T2		0.844 (0.679–0.973)	229.1	62.5	82.1	50	88.5
T3		0.817 (0.643–0.947)	199.6	75	57.1	33.3	88.9

*Note:* Data are presented as AUCs with bootstrap 95% CIs, cutoff values (pg/mL), and diagnostic indices for FGF21 at each time point. Bootstrap‐based internal validation was applied to the AUC estimates only; cutoff values and diagnostic performance measures were calculated from the original analytic dataset.

Abbreviations: AUC, area under the curve; CI, confidence interval; HOMA‐IR, homeostasis model assessment of insulin resistance; NPV, negative predictive value; PPV, positive predictive value.

Figure 5A shows the ROC curves for predicting postpartum glucose intolerance based on FGF21 concentrations measured at approximately 26 (T1) and 36 (T2) weeks of gestation and within 7 days postpartum (T3). In bootstrap‐based internal validation using 1000 stratified resampling iterations, the AUCs were 0.695 (95% CI, 0.498–0.867), 0.673 (95% CI, 0.476–0.860), and 0.606 (95% CI, 0.419–0.790) at T1, T2, and T3, respectively.

Figure 5B shows the predictive performance of FGF21 for postpartum insulin resistance. In bootstrap‐based internal validation, the AUCs were 0.871 (95% CI, 0.732–0.973), 0.844 (95% CI, 0.679–0.973), and 0.817 (95% CI, 0.643–0.947) at T1, T2, and T3, respectively.

### 3.4 Exploratory Subgroup Analyses According to Clinical Features of GDM

Additional subgroup analyses were performed within the GDM group according to insulin use during pregnancy and the timing of GDM diagnosis. Among all 45 women with GDM, FGF21 concentrations did not differ significantly between women who used insulin and those who did not at T1, T2, or T3. Similarly, no significant differences in FGF21 concentrations were observed between women diagnosed before 24 weeks 0 days of gestation and those diagnosed at 24 weeks 0 days of gestation or later at any time point (Table S1).

Among the 36 women with GDM who underwent a postpartum 75 g OGTT, postpartum metabolic outcomes were compared according to insulin use during pregnancy and the timing of GDM diagnosis (Table S2). Postpartum glucose intolerance and insulin resistance did not differ significantly according to insulin use or the timing of GDM diagnosis.

## 4 Discussion

This study yielded two key findings. First, serum FGF21 concentrations increased significantly from approximately 26 weeks (T1) to approximately 36 weeks (T2) and remained elevated within 7 days postpartum (T3), regardless of the GDM status. Second, perinatal FGF21 concentrations, particularly those measured at approximately 26 weeks of gestation, were associated with postpartum insulin resistance in women with GDM.

### 4.1 Changes in Serum FGF21 Concentrations

In this study, serum FGF21 concentrations were significantly higher in late pregnancy and the early postpartum period than in the late second trimester, regardless of the GDM status. This temporal pattern is consistent with findings by Mosavat et al. [9], who reported an increase from 24–28 weeks to delivery and additional increases within 24 h postpartum. In contrast, Li et al. [5] observed a decline from late pregnancy to 7 days postpartum.

Taken together with our findings based on blood samples obtained 1–7 days postpartum, it is plausible that FGF21 concentrations increase during late pregnancy and begin to decrease shortly after delivery. Our additional exploratory analyses showed significantly lower log1p‐transformed T3/T1 and T3/T2 ratios in samples collected later (Days 5–7) than in those collected earlier (Days 1–4), suggesting a decrease in postpartum FGF21 concentrations. This may partially explain the discrepancies in the literature and underscores the importance of the timing of sampling.

Furthermore, correlation analysis using continuous sampling day data revealed a significant inverse relationship between postpartum day and the log1p‐transformed T3/T1 ratio, further suggesting that FGF21 concentrations decrease soon after delivery. These findings reinforce the need for careful consideration of sampling timing in studies assessing FGF21 dynamics. Further longitudinal studies with more frequent sampling during the early postpartum period are warranted.

### 4.2 Analytical Focus on Longitudinal FGF21 Dynamics

Direct between‐group comparisons of absolute FGF21 concentrations at each time point were not the primary focus of this study. This decision was based on the substantial differences in the baseline characteristics between the GDM and control groups, particularly with respect to the pre‐pregnancy BMI, which is a determinant of circulating FGF21 concentrations. Thus, unadjusted comparisons at individual time points could be confounded by these background differences. Although FGF21 concentrations appeared to be higher in the control group at T2, this apparent difference should be interpreted cautiously because of the small sample size, interindividual variability, differences in background characteristics, sampling conditions, and possible unmeasured effects of treatment or lifestyle modifications after GDM diagnosis.

Instead, our analysis emphasized within‐group longitudinal changes in FGF21 concentrations and their association with postpartum metabolic outcomes, particularly in women with GDM. Notably, both groups demonstrated a similar temporal pattern, with FGF21 concentrations increasing from mid‐ to late‐pregnancy and remaining elevated during the early postpartum period. This parallel increase may reflect the physiological insulin resistance associated with normal pregnancy rather than GDM‐specific alterations, as reported by Catalano et al. [23]. This interpretation is further supported by earlier studies demonstrating positive correlations between circulating FGF21 levels and insulin resistance markers such as HOMA‐IR scores [2, 24, 25].

Previous studies comparing FGF21 levels between women with and without GDM have yielded inconsistent results, with some reporting higher levels in the GDM group [5, 6, 7, 8], others reporting higher levels in the controls [9], and others finding no significant differences [10, 11, 12, 13]. Meta‐analyses have suggested an overall tendency toward higher FGF21 levels in GDM, while also highlighting substantial heterogeneity related to the study design, population characteristics, and, importantly, the timing of blood sampling [14, 15]. Our findings underscore the importance of considering these factors when interpreting the between‐group differences.

To further address the clinical heterogeneity of GDM, we performed additional subgroup analyses according to insulin use and the timing of GDM diagnosis. These analyses did not show significant differences in FGF21 concentrations at any time point. Postpartum metabolic outcomes also did not differ significantly according to insulin use or the timing of diagnosis. These findings suggest that the overall pattern of FGF21 dynamics was not clearly explained by these markers of GDM severity in this cohort. However, because the subgroup analyses were limited by the small sample size, the influence of GDM heterogeneity on FGF21 dynamics and postpartum metabolic outcomes should be interpreted cautiously.

### 4.3 ROC Curves for Predicting Postpartum Glucose Intolerance and Insulin Resistance

In ROC analyses of women with GDM, FGF21 concentrations during pregnancy and the postpartum period showed varying predictive performance for postpartum glucose intolerance and insulin resistance. In particular, FGF21 concentrations measured at approximately 26 weeks showed the highest AUC for insulin resistance (AUC: 0.871). These findings suggest that FGF21 concentrations during the late second trimester may have potential as complementary indicators of postpartum metabolic risk in women with GDM.

However, these ROC findings should be interpreted as exploratory because of the limited sample size and the relatively wide bootstrap CIs. In addition, the diagnostic performance measures, including sensitivity, specificity, PPV, and NPV, varied across time points and outcomes, indicating that FGF21 alone is insufficient as a standalone screening test.

Postpartum assessment of glucose metabolism is crucial because women with GDM are at an increased risk of Type 2 diabetes [18, 19]. However, OGTT adherence remains low due to the patient burden [17, 20]. If FGF21 can help stratify risk using routine blood samples in combination with other clinical factors, it may help prioritize women for intensified postpartum follow‐up.

Future studies should focus on developing multivariate models that incorporate FGF21 and other clinical variables and validate their clinical utility. These models can inform individualized screening and postpartum surveillance strategies.

## 5 Limitations

This study has several limitations. First, the sample size was relatively small, particularly for analyses restricted to women with GDM who underwent postpartum OGTT, including the ROC analysis, which may have limited the statistical power to detect significant differences or ensure robust predictive performance. In addition, the group sizes of the GDM and control groups were unequal owing to the availability of preserved serum samples. Participants were selected based on the availability of preserved serum samples at all three time points and sufficient residual serum volume for FGF21 measurement; therefore, a selection bias may have been introduced. Although statistical tests appropriate for unequal sample sizes were applied, this imbalance and the selection process may still affect the generalizability of the intergroup comparisons.

Second, this study relied on blood samples obtained as part of routine clinical practice, and there was variability in the timing of sample collection. Postpartum blood samples were collected at a single time point between 1 and 7 days after delivery. Although additional exploratory analyses suggested that FGF21 concentrations may decline during the early postpartum period, the possibility of residual confounding related to postpartum sampling timing could not be fully excluded. Given the likelihood of rapid metabolic changes during early puerperium, more frequent and precise postpartum sampling is necessary to fully characterize the temporal trajectory of serum FGF21 concentrations. Moreover, sampling times at T3 systematically differed according to the mode of delivery, with earlier samples obtained from women who underwent vaginal delivery and later samples obtained from those who underwent cesarean sections. This imbalance may have introduced a potential confounding effect that could not be fully accounted for in this study. In addition, because circulating FGF21 levels have been reported to exhibit circadian variation and may be affected by fasting status [26], differences in sampling conditions between T1/T2 and T3 may have influenced the measured FGF21 concentrations.

Third, although previous studies demonstrated that circulating FGF21 levels are influenced by various factors, including dietary intake [27], physical activity [28], liver function [29], and lipid metabolism [3], the present study did not assess or adjust for these potential confounders. In addition, although the sensitivity analysis restricted to participants with a pre‐pregnancy BMI < 25 kg/m^2^ showed a longitudinal FGF21 pattern similar to that observed in the full cohort, residual confounding by BMI, particularly in the association between FGF21 concentrations and postpartum metabolic outcomes, could not be fully excluded. Consequently, residual confounding factors may have influenced the observed associations. Future studies incorporating measurements of lipid‐related metabolic markers and other relevant covariates are warranted to clarify the independent role of FGF21 in the regulation of perinatal metabolism.

Fourth, although we performed additional subgroup analyses according to insulin use and the timing of GDM diagnosis, the sample size of each subgroup was limited. Therefore, the influence of GDM heterogeneity and severity, including treatment modality and diagnostic timing, could not be fully evaluated. Future studies with larger sample sizes are needed to clarify whether FGF21 dynamics differ according to GDM severity or clinical subtype.

Finally, owing to the observational nature of this study, a causal relationship between perinatal FGF21 concentrations and postpartum metabolic outcomes could not be established.

In conclusion, our findings suggest that perinatal FGF21 concentrations may reflect physiological changes in insulin sensitivity during and after pregnancy, and that FGF21 measured at approximately 26 weeks of gestation may be associated with postpartum insulin resistance. Although preliminary, these findings contribute to the growing body of evidence regarding FGF21 as a potential supplementary indicator for postpartum metabolic risk assessment in women with GDM. Given the limited sample size, relatively wide bootstrap CIs, and lack of adjustment for key confounding factors, further large‐scale studies incorporating multivariate modeling and external validation are warranted to clarify the clinical utility of FGF21 before it can be considered for routine clinical use. At present, FGF21 should be regarded as a complementary indicator rather than a substitute for established screening methods such as the OGTT.

## Author Contributions

**Jun Okaniwa:** methodology, data curation. **Yuko Hasegawa:** methodology, data curation. **Akihito Morita:** conceptualization, methodology, investigation, writing – original draft, validation, writing – review and editing, supervision, visualization, formal analysis. **Yosuke Suzuki:** conceptualization, methodology, software, investigation, writing – original draft. **Akira Iwase:** funding acquisition, writing – review and editing, project administration. **Ayuko Tanaka:** methodology, data curation. **Daisuke Higeta:** methodology, data curation. **Tatsuya Sato:** methodology, data curation.

## Ethics Statement

This study was approved by the Ethics Committee of Gunma University Graduate School of Medicine (approval number: HS2018‐214; approval date: February 2, 2019).

## Consent

Written informed consent was obtained from all participants prior to inclusion in the study.

## Conflicts of Interest

The authors declare no conflicts of interest.

## Supporting information


**File S1:** De‐identified individual‐level dataset used for the analyses in this study.

**Table 1** Serum FGF21 concentrations according to insulin use and timing of GDM diagnosis.

**Table 2** Postpartum metabolic outcomes in women with GDM who underwent postpartum oral glucose tolerance test.

**Figure S1:** Longitudinal changes in serum FGF21 concentrations in women with GDM and controls with a pre‐pregnancy BMI < 25 kg/m^2^.
Serum FGF21 concentrations at approximately 26 (T1) and 36 (T2) weeks of gestation and within 7 days postpartum (T3) in participants with a pre‐pregnancy BMI < 25 kg/m^2^ (GDM, *n* = 20; control, *n* = 26). Box plots show the median, interquartile range, and whiskers extending to 1.5 × IQR from the quartiles. *p*‐values were obtained using Wilcoxon signed‐rank tests and adjusted for multiple comparisons using the Benjamini–Hochberg false discovery rate method following a significant Friedman tests.

## Data Availability

The de‐identified individual‐level dataset used for the analyses in this study is provided as File S1.
